# Effects of different types of Ringer’s solution on patients with traumatic haemorrhagic shock: a prospective cohort study

**DOI:** 10.1186/s40001-024-01664-3

**Published:** 2024-04-03

**Authors:** Qing Li, Qiang Yang, Chao Tian, Yao Guo, Hui Liu, Yadong Cheng, Shu-Zhen Bi, Jin-Hua Chen

**Affiliations:** Department of Emergency, Changzhi People’s Hospital, No. 502 of Changxing Street, Luzhou District, Changzhi, 046000 China

**Keywords:** Traumatic haemorrhagic shock, Sodium bicarbonate Ringer’s solution, Sodium acetate Ringer’s solution, Fluid resuscitation

## Abstract

**Objective:**

To compare the fluid resuscitation effect of sodium acetate Ringer’s solution and sodium bicarbonate Ringer’s solution on patients with traumatic haemorrhagic shock.

**Method:**

We conducted a prospective cohort study in our emergency department on a total of 71 patients with traumatic haemorrhagic shock admitted between 1 December 2020 and 28 February 2022. Based on the time of admission, patients were randomly divided into a sodium bicarbonate Ringer’s solution group and sodium acetate Ringer’s solution group, and a limited rehydration resuscitation strategy was adopted in both groups. General data were collected separately, and the patients’ vital signs (body temperature, respiration, blood pressure and mean arterial pressure (MAP)), blood gas indices (pH, calculated bicarbonate (cHCO_3_^−^), partial pressure of oxygen (PaO_2_), partial pressure of carbon dioxide (pCO_2_) and clearance of lactate (CLac)), shock indices, peripheral platelet counts, prothrombin times and plasma fibrinogen levels were measured and compared before and 1 h after resuscitation.

**Results:**

The post-resuscitation heart rate of the sodium bicarbonate Ringer’s solution group was significantly lower than that of the sodium acetate Ringer’s solution group (*p* < 0.05), and the MAP was also significantly lower (*p* < 0.05). The patients in the sodium bicarbonate Ringer’s solution group had significantly higher pH, cHCO_3_^−^ and PaO_2_ values and lower pCO_2_ and CLac values (*p* < 0.05) than those in the sodium acetate Ringer’s solution group, and the post-resuscitation peripheral platelet counts and fibrinogen levels were significantly higher, with shorter plasma prothrombin times and smaller shock indices (*p* < 0.001).

**Conclusion:**

Sodium bicarbonate Ringer’s solution is beneficial for maintaining MAP at a low level after resuscitation. The use of sodium bicarbonate Ringer’s solution in limited fluid resuscitation has positive results and is of high clinical value.

## Introduction

During traumatic haemorrhagic shock, the effective circulating blood volume is severely insufficient, leading to tissue hypoperfusion, cellular hypoxia, tissue damage and, ultimately, death [1]. Fluid resuscitation is a key component of early treatment in traumatic haemorrhagic shock [2]. Emergency surgeons in hospitals may encounter patients with this condition on a daily basis and must open a liquid route and make a clear diagnosis as soon as possible during the emergency stage of treatment. The guidelines require the infusion of blood products to patients with traumatic haemorrhagic shock within half an hour of the trauma [3]; therefore, the ability to select a fluid for resuscitation at short notice has significant clinical value. The Advanced Trauma Life Support Group of the American College of Surgeons recommends the use of an equilibrium fluid, such as Ringer’s solution, as the first-line fluid for the fluid resuscitation of traumatic shock [4]. However, the most effective type of Ringer’s solution to use continues to be debated. Han et al. [5] compared the efficacy of sodium bicarbonate Ringer's solution and sodium lactate Ringer's solution in the treatment of traumatic liver rupture with haemorrhagic shock. The results showed that compared with sodium lactate Ringer's solution, sodium bicarbonate Ringer's solution could significantly reduce the occurrence of shock-related complications, inhibit the expression of inflammatory factors in peripheral blood and correct acidosis. Wang et al. [6] compared the therapeutic effects of sodium acetate Ringer solution and sodium lactate Ringer solution on patients with traumatic hemorrhagic shock. The results showed that the early use of sodium acetate Ringer solution in patients with traumatic hemorrhagic shock could better inhibit the expression of inflammatory factors in peripheral blood and further reduce the level of Lac in patients with traumatic haemorrhagic shock. Therefore, comparing the efficacy of different kinds of Ringer's solution in patients with traumatic haemorrhagic shock is helpful to make a more effective clinical treatment plan.

Sodium acetate Ringer’s solution is highly recommended for the following reasons: I. acetic acid metabolism is fast and does not depend on liver and kidney metabolism; II. it can enhance immune function, which is conducive to maintaining the body's core temperature under general anaesthesia [7]. However, the use of sodium bicarbonate Ringer’s solution is also commonly recommended. It is the closest physiological equilibrium liquid to plasma, with a comprehensive ionic composition similar to that of plasma. Furthermore, it has a physiological bicarbonate (HCO_3_^−^) buffer system that can quickly produce an alkali effect without requiring the body’s metabolic processes and can alleviate acidosis [8]; moreover, it does not contain lactate nor interfere with the use of lactate to rapidly replenish circulating blood volume, which is conducive to improving tissue perfusion, increasing blood and oxygen supply, restoring haemodynamic stability, and maintaining the balance of water, electrolytes, osmolarity pressure, acids and bases [9].

This study aims to provide emergency surgeons with an enhanced resuscitation regimen when treating patients with traumatic haemorrhagic shock. It explores the effect of sodium bicarbonate Ringer’s solution and sodium acetate Ringer’s solution on liquid resuscitation in the emergency stage of traumatic haemorrhagic shock.

## Data and methods

### Population

Between 1 December 2020 and 28 February 2022, we selected patients who were admitted to our hospital’s emergency department based on the following criteria:

Inclusion criteria: (1) a diagnosis of haemorrhagic shock with comprehensive clinical evaluation; (2) an injury time of ≤ 6 h; (3) the provision of informed consent by the patient or their family. Exclusion criteria: (1) death within 1 h of admission; (2) organic lesions of vital organs; (3) a history of previous coagulopathy.

### Methods

For simple randomisation, we divided 71 patients according to the chronological order by date of their admission to hospital.

For the sodium bicarbonate Ringer’s solution group, the specific measures were as follows. The patients’ vital signs were monitored immediately after admission, and their bedside blood gas levels were checked and recorded. Once traumatic haemorrhagic shock was confirmed, two venous accesses were initially opened; medical staff then evaluated the blood loss of the patients in shock. Sodium bicarbonate Ringer’s solution (Jiangsu Hengrui, Lianyungang, China) was infused in one of the venous accesses at 1–1.5 mL/(kg/min) within the first 30 min of anti-shock treatment; the infusion volume and speed were adjusted according to the patient's blood pressure and heart rate so that the systolic blood pressure rose to 10–12 kPa (75–90 mmHg), and then blood pressure was maintained. If haematocrit was < 30%, a blood transfusion was given. After infusion, the patients were given 500 mL of 0.9% sodium chloride solution.

For the sodium acetate Ringer’s solution group, sodium acetate Ringer’s solution (Hubei Dorui, China) was infused in one of the venous accesses. All other operations, such as infusion speed and volume, were the same as for the sodium bicarbonate Ringer’s solution group.

### Observation indicators

In both groups, 5 mL of peripheral venous blood, 1 mL of arterial blood and 1 mL of central venous blood were collected before limited fluid resuscitation and 1 h after resuscitation. A blood gas analysis of the peripheral venous blood, arterial blood and central venous blood was performed using an arterial blood analyser. The arterial blood lactate value and venous partial pressure of oxygen of the sample were recorded.

General data and vital signs before and 1 h after resuscitation were collected from both groups, including body temperature, heart rate, breathing rate, systolic blood pressure, diastolic blood pressure, mean arterial pressure (MAP), blood gas index (pH, partial pressure of carbon dioxide (pCO_2_), calculated bicarbonate (cHCO_3_^−^) and clearance of lactate (CLac)), shock index, peripheral platelet count, plasma prothrombin time and fibrinogen level.

### Statistical analysis

A statistical analysis was performed using SPSS 26.0 software (v. 24.0). Quantitative data were presented as mean ± standard deviation (x ± s), and an independent-samples *t* test was used for comparisons between the two groups. Qualitative data were presented as *n*(%), and a comparison of the two groups was performed using the Chi-squared test. A value of *p* < 0.05 indicated a statistically significant difference.

## Results

### Comparison of various clinical baseline data between the two groups

The patients were aged 15–80 years, with a mean of 49.91 ± 12.91 years. There were 59 men and 12 women. There were no significant differences in age, sex, abbreviated injury scale score, retention time and fluid infusion volume between the two groups (*p* > 0.05)(see Table 1 for details).

**Table 1:** Comparison of clinical baseline data before the resuscitation between the two groups

Item	Sodium bicarbonate ringer liquid group	Sodium acetated ringer liquid group	*t*/*x*^2^	*P*
Age	50.430 ± 1.850	49.330 ± 2.559	0.353	0.725
Gender			0.287	0.592
Male	32 EEE(86.5)	27 (79.4)		
Female	5 (13.5)	7 (20.6)		
AIS Index score				0.846
2	1 (2.7)	1 (3.0)		
3	3 (8.1)	5 (14.7)		
4	29 (78.4)	25 (73.5)		
5	4 (10.8)	3 (8.8)		
Fluid infusion volume	969.732 ± 62.520	1043.946 ± 76.802	−0.756	0.452
Retention time	30.574 ± 3.861	30.458 ± 4.864	0.018	0.985

### Comparison of vital sign indicators of the two groups before and after resuscitation

There were no significant differences in vital signs before resuscitation between the sodium bicarbonate Ringer’s solution group and sodium acetate Ringer’s solution group (*p* > 0.05).Compared with pre-resuscitation, MAP and body temperature in both groups was significantly higher (*p* < 0.05), and their heart rate and breathing rate were decreased 1 h after resuscitation. The difference was statistically significant (*p* < 0.05). Compared with the post-resuscitation vital sign indicators of the sodium acetate Ringer’s solution group, the post-resuscitation heart rate of the sodium bicarbonate Ringer’s solution group was significantly reduced (92.790 ± 16.355 vs 86.220 ± 16.063, *p* < 0.05) and the MAP was also significantly reduced (87.706 ± 16.010 vs 83.214 ± 16.278, *p* < 0.05) (see Table 2 for details).

**Table 2:** Comparison of vital signs indicators before and after resuscitation

Observation items	Sodium bicarbonate ringer liquid group		Sodium acetated ringer liquid group	
	Before resuscitation	After resuscitation	Before resuscitation	After resuscitation
Body temperature (℃)	36.368 ± 0.4899	37.205 ± 3.304*	36.462 ± 0.665	36.465 ± 0.659*
Heart rate (beat/min)	95.670 ± 19.016	86.220 ± 16.063*^#^	96.650 ± 21.621	92.790 ± 16.355*
Breathing rate (time/min)	19.570 ± 4.764	18.430 ± 3.468*	19.182 ± 4.417	18.912 ± 2.404*
MAP (mmHg)	82.683 ± 22.683	83.214 ± 16.278*^#^	86.200 ± 21.734	87.706 ± 16.010*

Within the same group, compared with that before resuscitation, *p < 0.05, **p < 0.001

Within different groups, comparing in the same state, ^#^*p* < 0.05

### Comparison of blood gas indices of the two groups before and after resuscitation

There were no significant differences in pH, pCO_2_ or cHCO_3_^−^ indicators before resuscitation between the two groups (*p* > 0.05), and the differences in pCO_2_ and CLac were not significant before resuscitation (*p* > 0.05). Compared with before resuscitation, after resuscitation, the sodium bicarbonate Ringer’s solution group exhibited significantly higher pH and cHCO_3_^−^ (*p* < 0.05), lower pCO_2_ (37.549 ± 16.250 vs 35.862 ± 12.476, *p* < 0.05), higher mixed venous oxygen tension (PvO_2_; 71.357 ± 23.155 vs 85.114 ± 22.098, *p* < 0.001) and lower CLac (3.336 ± 2.879 vs 2.068 ± 2.2065, *p* < 0.05) values. After resuscitation, the pH, pCO_2_ and cHCO_3_^−^ values in the sodium acetate Ringer’s solution group were increased; however, the difference was not statistically significant. In the sodium acetate Ringer’s solution group, the PvO_2_ value was significantly increased (70.753 ± 20.679 vs 96.050 ± 71.579, *p* < 0.05) and CLac value was significantly decreased (3.622 ± 4.146 vs 3.155 ± 4.944, *p* < 0.05).

Compared with the various blood gas indices of the sodium acetate Ringer’s solution group after resuscitation, the patients in the sodium bicarbonate Ringer’s solution group had higher post-resuscitation pH, cHCO_3_^−^ and partial pressure of oxygen (PaO_2_) values and lower pCO_2_ and CLac values. The differences were all significant (*p* < 0.05) (see Table 3 for details).

**Table 3:** Comparison of blood gas indexes before and after resuscitation between the two groups

Observation indicators	Sodium bicarbonate ringer liquid group		Sodium acetated ringer liquid group	
	Before resuscitation	After resuscitation	Before resuscitation	After resuscitation
pH	7.369 ± 0.127	7.392 ± 0.092^**#^	7.345 ± 0.095	7.351 ± 0.116
PCO_2_	37.549 ± 16.250	35.862 ± 12.476^*#^	34.256 ± 7.730	37.009 ± 9.378
CHCO_3_^−^	21.035 ± 3.937	21.954 ± 3.681^*#^	19.441 ± 4.423	20.900 ± 4.3341
PvO_2_	71.357 ± 23.155	85.114 ± 22.098^**#^	70.753 ± 20.679	96.050 ± 71.579^*^
Lac	3.336 ± 2.879	2.068 ± 2.2065^*#^	3.622 ± 4.146	3.155 ± 4.944^*^

Within the same group, compared with that before resuscitation, *p < 0.05, **p < 0.001

Within different groups, comparing in the same state, ^#^*p* < 0.05

### Comparison of blood routine and laboratory indicators of the two groups before and after resuscitation

The pre-resuscitation shock index, peripheral platelet count, plasma prothrombin time and fibrinogen level of the two groups were not significantly different (*p* > 0.05). In both groups, compared with pre-resuscitation, the shock index, peripheral platelet count and fibrinogen level were significantly lower and the plasma prothrombin time was significantly higher (*p* < 0.05) after resuscitation.

Compared with the indicators in the sodium acetate Ringer’s solution group, the peripheral platelet count and fibrinogen level were higher, plasma prothrombin time shorter and shock index smaller (*p* < 0.05) in the sodium bicarbonate Ringer’s solution group after resuscitation (see Table 4 for details).

**Table 4:** Comparison of blood routine and laboratory indicators between the two groups before and after resuscitation

Indicators	Sodium bicarbonate ringer liquid group		Sodium acetated ringer liquid group	
	Before resuscitation	After resuscitation	Before resuscitation	After resuscitation
Shock index	0.939 ± 0.355	0.793 ± 0.211*^#^	0.902 ± 0.289	0.823 ± 0.2867*
Peripheral platelet count (109/L)	180.034 ± 3.321	156.623 ± 1.340*^#^	185.752 ± 3.426	142.153 ± 1.528*
Plasma prothrombin time (s)	20.292 ± 3.254	21.975 ± 4.117*^#^	20.759 ± 3.023	24.456 ± 3.951*
Fibrinogen level (g/L)	4.295 ± 2.194	3.684 ± 1.731*^#^	4.032 ± 1.845	3.145 ± 2.427*

Within the same group, compared with that before resuscitation, *p < 0.05, **p < 0.001

Within different groups, comparing in the same state, ^#^*p* < 0.05

## Discussion

Traumatic haemorrhagic shock is a systemic disease caused by body metabolic disorders and a systemic inflammatory environment [7]. Between 66 and 80% of traumatic deaths are caused by haemorrhagic shock, and the first task of treating the condition is to manage the cause of bleeding and immediately restore blood loss [10]. Patients with traumatic shock entering trauma centres or undergoing emergency surgery should receive shock resuscitation within 1 h of admission, followed by surgical, interventional or other treatments [11]. Therefore, this study chose 1 h after resuscitation as the time window for comparison, during which the cause of bleeding is treated as early as possible to effectively prevent continued blood loss.

Because different treatment methods are incomparable and significantly impact the cure rate of patients, this study focused only on the different effects of the two equilibrium fluids on vital signs, blood gas, blood routine and laboratory indicators 1 h after resuscitation to evaluate the resuscitation effect of the different fluids.

Whether to choose a crystalline solution or colloidal solution for resuscitation fluids has been a focus of debate. At present, the academic community views the application of artificial colloidal fluids in patients with traumatic haemorrhagic shock negatively because the infusion of excessive colloidal fluids, such as hydroxyethyl starch, can improve tissue oedema but may cause some damage to patients’ renal function [12]. Therefore, the preference for crystalline fluids has been widely recognised and recommended in early fluid resuscitation treatment in patients with traumatic haemorrhagic shock.

Furthermore, haemorrhagic shock is closely related to the occurrence of metabolic acidosis. The extensive use of normal saline for resuscitation easily leads to perchlorinated metabolic acidosis. Because chloride can be partially replaced by metabolisable anions, such as lactate, acetate or malate, the use of sodium acetate Ringer’s solution, sodium lactate Ringer’s solution or bicarbonate Ringer’s solution is mostly recommended when selecting isotonic crystals [13, 14].

Limited fluid resuscitation has been widely cited as a new concept. It refers to the notion that in non-controlled haemorrhagic shock, by controlling the speed and volume of liquid infusion, the patient's blood pressure can be maintained at a lower or normal level to guarantee blood supply to vital organs until bleeding is stopped completely, enabling the compensatory mechanism of the body and resuscitation effect of the fluid to be fully engaged to achieve the ideal resuscitation effect [15]. The concept advocates using a small volume of sodium acetate Ringer’s solution, sodium lactate Ringer’s solution or sodium bicarbonate Ringer’s solution and other fluids to maintain the basic needs of the vital organs before haemostasis, followed by full resuscitation after complete haemostasis. In this study, limited fluid resuscitation was used in both the sodium bicarbonate Ringer’s solution group and sodium acetate Ringer’s solution group.

Sodium bicarbonate Ringer's solution is weakly alkaline, with a pH of 7.3. It contains sodium, potassium, magnesium and calcium ions and can maintain the physiological activity of cells. The HCO_3_^−^ buffering system helps to reduce damage from acidosis without placing additional burdens on the kidneys and liver [16, 17].

Studies have demonstrated that the alkalisation effect of sodium bicarbonate Ringer’s solution is more pronounced than that of sodium acetate Ringer’s solution in partially hepatectomised rabbits, providing effective magnesium stability. The electrolyte ratio of sodium bicarbonate Ringer’s solution is relatively close to that of plasma, and it is mainly used for the correction of extracellular fluid supplementation when circulating blood flow and inter-tissue fluid are reduced as well as for the correction of metabolic acidosis [2, 18].

Mean arterial pressure is a traditional indicator of metabolic disorder in the post-shock period [9]. In this study, compared with the values in the sodium acetate Ringer’s solution group, both the heart rate and MAP in the sodium bicarbonate Ringer’s solution group were significantly lower, indicating that those patients with haemorrhagic shock who received sodium bicarbonate Ringer’s solution had more stable haemodynamics than those who received sodium acetate Ringer's solution and that sodium bicarbonate Ringer’s solution maintained MAP at a lower level after resuscitation.

Studies have revealed that during limited fluid resuscitation, maintaining MAP at 40 mmHg with compound Ringer’s solution results in a higher final survival rate compared with maintaining MAP at 80 mmHg. However, acidosis is significantly less severe at a MAP of 60 mmHg than at 40 mmHg [19]. Some scholars have proposed that MAP should be maintained at 40–50 mmHg, which is slightly higher than the minimum value required for survival. A ‘moderate amount’ is considered appropriate for the fluid resuscitation of haemorrhagic shock [20], but it is difficult to determine that amount in actual clinical practice because it is affected by the severity of the injury, type of resuscitation fluid and age of the patient. The MAP after resuscitation in our study was at a high level, which may be related to the infusion volume, heart rate or PvO_2_ value. Therefore, appropriate MAP targets require further investigation [21–23].

The results of our study revealed that the pH and HCO_3_^−^ values (*p* < 0.001) increased significantly after resuscitation in the sodium bicarbonate Ringer’s solution group. The possible reason for this is that the bicarbonate ion has a direct alkalising effect, it can correct acidosis through chemical neutralisation and it does not need to go through the series of metabolic processes required by lactic acid Ringer’s solution and acetic acid Ringer’s solution. The sodium acetate Ringer’s solution group displayed the same trend.

In haemorrhagic shock, the body’s circulation cannot provide enough oxygen to meet the needs of tissues, leading to cellular hypoxia and cellular dysfunction. As a result, oxygen delivery and oxygen consumption lose their physiological independence. After trauma, the massive loss of blood and inadequate tissue perfusion cause anaerobic enzymatic digestion to replace aerobic respiration, resulting in the production of large volumes of lactic acid [24]. In this study, we determined that the CLac value decreased significantly in both groups after resuscitation. Notably, compared with patients in the sodium acetate Ringer’s solution group, those in the sodium bicarbonate Ringer’s solution group had lower CLac values after resuscitation (p < 0.05). Although the acetic acid provided by sodium acetate Ringer’s solution is rapidly metabolised to acetyl coenzyme A, acetyl coenzyme A condenses with oxaloacetate to form citric acid, which passes through the tricarboxylic acid cycle to form bicarbonate. Within 1 h, sodium bicarbonate Ringer’s solution, through its direct provision of bicarbonate, can lower a patient's CLac even more significantly [25]. However, we noted that patients treated with sodium bicarbonate Ringer’s solution had significantly higher PvO_2_ values, consistent with the results of previous clinical trials [26, 27].

Moreover, sodium bicarbonate Ringer’s solution was able to inhibit the occurrence of the inflammatory response. A study demonstrated that the release of inflammatory factors, such as tumour necrosis factor α (TNF⁃α) and interleukin 6 (IL-6), is significantly increased after trauma and can be used as an indicator to assess the trauma severity of patients and for clinical prognosis [28]. Previous studies have compared the limited resuscitation effect of sodium bicarbonate Ringer’s solution with sodium lactated bicarbonate Ringer’s solution, demonstrating that patients treated with sodium lactated bicarbonate Ringer’s solution had higher inflammatory factor levels than those treated with sodium bicarbonate Ringer’s solution [9]. Animal experiments revealed that the infusion of sodium lactate Ringer’s solution induced oxidative stress in neutrophils, promoted the release of inflammatory factors, such as TNF⁃α and IL⁃6, and aggravated the inflammatory response [7]. Because the CLac value was lower in the sodium bicarbonate Ringer’s group than in the sodium acetate Ringer’s solution group, we can speculate that sodium bicarbonate Ringer’s solution controls lactate levels in the body more effectively than sodium acetate Ringer’s solution and thus can suppress the inflammatory response of the body after trauma. However, this conclusion needs to be confirmed through more in-depth clinical studies.

In addition, in both groups, the results of this study revealed that compared with pre-resuscitation, the peripheral platelet count and fibrinogen level were significantly decreased, and the plasma prothrombin time was significantly higher (*p* > 0.05). However, the post-resuscitation peripheral platelet count and fibrinogen level in the sodium bicarbonate Ringer’s solution group were higher than those in the sodium acetate Ringer’s solution group, and the plasma prothrombin time was shorter. Although limited fluid resuscitation increases fibrinogen degradation and prolongs the blood coagulation time, which may lead to blood coagulation dysfunction, the use of sodium bicarbonate Ringer’s solution controlled the decrease in both the peripheral platelet count and fibrinogen levels and shortened the plasma prothrombin time compared with the use of sodium acetate Ringer’s solution. This suggests, at least partly, that the use of sodium bicarbonate Ringer’s solution in limited fluid resuscitation has stronger results and higher clinical value than the use of sodium acetate Ringer’s solution.

## Limitation

This study conducted a direct comparison of sodium bicarbonate Ringer’s solution and sodium acetate Ringer’s solution in terms of the limited fluid resuscitation effect in patients with haemorrhagic shock; however, it has some limitations. First, because of time limitations, we collected data from only 71 participants, and the conclusions obtained still need to be validated on a larger scale. Second, we used a simple randomisation method to divide the patients into two groups based on different types of Ringer’s solution, without using stratification to exclude some factors that may affect prognosis. Finally, future experiments should clarify the relationship between biological processes and prognosis.

## Conclusion

This study demonstrated that the post-resuscitation heart rate of the sodium bicarbonate Ringer’s solution group was significantly lower than that of the sodium acetate Ringer’s solution group, and their MAP was also significantly lower. The patients in the sodium bicarbonate Ringer’s solution group had significantly higher pH, cHCO3 − and PaO2 values and lower pCO2 and CLac values (*p* < 0.05) than those in the sodium acetate Ringer’s solution group, and the post-resuscitation peripheral platelet counts and fibrinogen levels were significantly higher, with shorter plasma prothrombin times and smaller shock indices (*p* < 0.001). Therefore, sodium bicarbonate Ringer’s solution is beneficial for maintaining MAP at a low level after resuscitation. The use of sodium bicarbonate Ringer’s solution in limited fluid resuscitation has positive results and is of high clinical value.

## Data Availability

All data generated or analyzed during this study are included in this published article.
